# Glycosaminoglycan profiles of repair tissue formed following autologous chondrocyte implantation differ from control cartilage

**DOI:** 10.1186/ar2278

**Published:** 2007-08-14

**Authors:** Aarti Sharma, Lindsay D Wood, James B Richardson, Sally Roberts, Nicola J Kuiper

**Affiliations:** 1 Institute of Science & Technology in Medicine (ISTM), University of Keele, Staffordshire, ST5 5BG, UK; 2 Institute of Orthopaedics, Robert Jones & Agnes Hunt (RJAH) Orthopaedic Hospital, ISTM, University of Keele, Oswestry, Shropshire, SY10 7AG, UK; 3 Centre for Spinal Studies, RJAH Orthopaedic Hospital, ISTM, University of Keele, Oswestry, Shropshire, SY10 7AG, UK

## Abstract

Currently, autologous chondrocyte implantation (ACI) is the most commonly used cell-based therapy for the treatment of isolated femoral condyle lesions of the knee. A small number of centres performing ACI have reported encouraging long-term clinical results, but there is currently a lack of quantitative and qualitative biochemical data regarding the nature of the repair tissue. Glycosaminoglycan (GAG) structure influences physiological function and is likely to be important in the long-term stability of the repair tissue. The objective of this study was to use fluorophore-assisted carbohydrate electrophoresis (FACE) to both quantitatively and qualitatively analyse the GAG composition of repair tissue biopsies and compare them with age-matched cadaveric controls. We used immunohistochemistry to provide a baseline reference for comparison. Biopsies were taken from eight patients (22 to 52 years old) 1 year after ACI treatment and from four cadavers (20 to 50 years old). FACE quantitatively profiled the GAGs in as little as 5 μg of cartilage. The pattern and intensity of immunostaining were generally comparable with the data obtained with FACE. In the ACI repair tissue, there was a twofold reduction in chondroitin sulphate and keratan sulphate compared with age-matched control cartilage. By contrast, there was an increase in hyaluronan with significantly shorter chondroitin sulphate chains and less chondroitin 6-sulphate in repair tissue than control cartilage. The composition of the repair tissue thus is not identical to mature articular cartilage.

## Introduction

Autologous chondrocyte implantation (ACI), using cultured chondrocytes implanted beneath a periosteal patch, is widely used to treat cartilage defects [1]. To date, only a small number of centres, including our own, have investigated the long-term stability of the repair tissue by means of outcome measures such as mechanical testing, clinical scores, magnetic resonance imaging, and limited histology [2-8]. Although the results have been encouraging, they have not determined the compositional changes of the repair tissue which are known to have major influences on its physiological function. Detailed biochemical analyses of the repair tissue and their comparison with native tissue are important considerations.

Glycosaminoglycans (GAGs) are a cartilage component with important physiological functions. With the exception of hyaluronan (HA), GAGs are synthesised covalently bound to core proteins to form proteoglycans such as aggrecan [9]. GAGs endow the tissue with resistance to compressive loading [10,11] and are involved in many biological interactions [12,13]. Chondroitin sulphate (CS), keratan sulphate (KS), and HA are three classes of GAGs found in articular cartilage. As a function of development, age, and disease, these GAGs exhibit changes in chain length, chain termination, sulphate ester substitution, and substitution of CS chains for KS chains on the aggrecan protein core [14-21]. Approximately 80% of GAGs in adult articular cartilage are CS chains. They are composed of glucuronic acid (GlcA) and *N*-acetylgalactosamine (GalNAc) which may be sulphated at the C2 site of GlcA and the C4 and/or C6 sites of GalNAc. The sugars are linked by a β1,3-glycosidic bond, and the chain is capped with a GalNAc residue that is usually sulphated [21]. The ratio of 6-sulphation to 4-sulphation in CS chains changes with development and age [14]. For example, foetal CS chains are equally 4- and 6-sulphated but adult CS chains are highly 6-sulphated. To add to the complexity of the CS chain, chain size and the terminal GalNAc may alter too [21]. Foetal CS chains are approximately 25 kD and almost entirely capped with a 4-sulphated GalNAc, whereas adult CS chains are approximately 16 kD and mostly capped with a 4- and 6-sulphated GalNAc [22]. These alterations in sulphation, chain length, and chain termination may have roles in directing the location or activities of extracellular matrix proteins.

KS represents 5% to 20% of the GAG chains in articular cartilage. KS is composed of galactose (Gal) and *N*-acetylglucosamine (GlcNAc) linked by a β1,4-glycosidic bond [23,24]. KS chains can be modified by O-sulphation of the hydroxyl groups but in a much more restricted pattern when compared with CS. O-sulphation of the hydroxyl groups at the C6 site of both the Gal and/or the GlcNAc results in un-, mono-, or disulphated repeat disaccharides [25]. KS chains demonstrate an age-related increase in chain length and sulphation [23-25].

HA accounts for 1% to 10% of GAGs in articular cartilage and is composed of GlcA and GlcNAc linked by a β1,3-glycosidic bond. Studies investigating the changes in HA in articular cartilage during maturation and ageing are limited. One study, using a radiosorbent assay with large quantities of whole tissue, reported that the molecular mass of HA decreased and the concentration increased with increasing age [20]. High concentrations of HA are generally found during development and the early stages of wound healing and repair [26,27].

To date, the assessment of the proteoglycan and GAG composition of ACI repair tissue has been mainly by metachromasia or immunolocalisation techniques [2-5]. Metachromasia has provided a crude assessment of the repair tissue with a limited amount of information [2,5]. Immunolocalisation techniques have provided some information about the type and distribution of GAGs (mainly KS and CS) but little information about their chemistry or quantitation [3,4]. Biochemical analyses will always be restricted by the limited repair tissue available in humans and the small biopsy size (250 μg). Consequently, it would be advantageous to have a technique that could analyse GAG content sensitively in small samples of tissue. At present, the methods available for analysing GAGs are generally crude and require large amounts of starting material (5 to 100 mg) [18-20,28-30]. The dimethylmethylene blue assay is widely used for the quantitation of total sulphated GAGs [28], but it does nothing to differentiate between types of GAGs and it can also be inaccurate due to interference from polyanions from other sources. Other techniques, such as the uronic acid assay [29], provide little information about the GAG population and can be time-consuming. Fluorophore-assisted carbohydrate electrophoresis (FACE) has evolved recently to be a highly sensitive technique for profiling GAGs in small amounts of tissue and body fluids [13,31-34] and has the added advantage of being able to process a number of samples simultaneously. As an analytical technique, FACE [13] can provide more information about GAGs than other techniques such as radiolabeling [30], nuclear magnetic resonance [21], or high-performance liquid chromatography [18]. Consequently, FACE could complement existing immunolocalisation techniques.

The aim of this study was to use FACE to both quantitatively and qualitatively analyse the GAG composition of ACI repair tissue biopsies and compare them with age-matched cadaveric controls. We have used immunohistochemistry to provide a baseline reference for comparison.

## Materials and methods

### Biopsies of autologous chondrocyte implantation repair tissue

In our centre, patients with focal chondral defects of either the medial femoral condyle or the lateral femoral condyle received ACI using cultured chondrocytes implanted beneath a periosteal patch [1]. Patients underwent arthroscopy and biopsy of the treated region as part of their routine follow-up at approximately 1 year post-ACI [3,4]. A single biopsy (1.8 mm in diameter and approximately 5 mm in height) was taken perpendicularly from the articulating surface through the full depth of cartilage and subchondral bone from each of eight patients 1 year post-ACI (Table 1) using a detailed knee map to identify the repair site. The removal of biopsies from ACI-treated patients was given ethical approval by the Shropshire Research and Ethics Committee (UK). All patients gave fully informed consent.

**Table 1:** Patient age and the site and size of the chondral defect treated with autologous chondrocyte implantation

Patient age (years)	Site of autologous chondrocyte implantation	Defect size (mm)
22	Medial femoral condyle	20 × 30
25	Lateral femoral condyle	27 × 21
25	Lateral femoral condyle	16 × 13
27	Lateral femoral condyle	30 × 40
42	Medial femoral condyle	14 × 12
44	Medial femoral condyle	18 × 15
45	Medial femoral condyle	25 × 8
52	Medial femoral condyle	21 × 13

### Biopsies of cadaveric control tissue

Healthy cadaveric knees (22, 30, 40, and 50 years old) were obtained within 24 hours of death from the UK Human Tissue Bank with approval by the Trent Research Ethics Committee (UK). All appeared macroscopically normal apart from the 50-year old, whose articular cartilage showed slight surface fibrillation. Biopsies were taken from each of the medial and lateral femoral condyles representative of regions most commonly treated with ACI, as described in the previous paragraph.

### Treatment of all biopsies and statistical analysis

All biopsies were snap-frozen in liquid nitrogen-cooled hexane and stored in liquid nitrogen until studied. Half of each biopsy was cryosectioned (10-μm sections) for histology and immunohistochemistry. The other half of each biopsy (approximately 250 μg) was digested, divided into eighths, and then analysed separately by FACE. Results for each biopsy were pooled. Where appropriate, results are presented as the mean ± the standard error of the mean. Statistical differences between the repair and control tissues were analysed using the Mann-Whitney test (significance level, *p *≤ 0.05).

### Preparation of glycosaminoglycan saccharides for FACE

The GAGs were extracted from repair tissue and control biopsies by means of a previously published methodology [31]. Purified GAGs were depolymerised using GAG-specific enzymes (Seikagaku Corporation, Tokyo, Japan). HA was digested into disaccharides (ΔDiHA) using 100 mU/ml of hyaluronidase (from *Streptococcus dysgalactiae*) for 1 hour at 37°C [31,32]. CS was digested into disaccharides (6-sulphated [ΔDi6S], 4-sulphated [ΔDi4S], and unsulphated [ΔDi0S]) with 100 mU/ml of chondroitinase ABC (cABC) for 3 hours at 37°C. Sulphation of CS was confirmed by incubation of cABC-digested samples with 100 mU/ml of chondroitin-4ase and/or chondroitin-6ase for 12 hours at 37°C. The nonreducing terminal sugars of CS were identified by mercuric ion treatment [31]. KS was digested as previously described [13]. Briefly, samples were incubated with 100 mU/ml of keratanase II for 3 hours at 37°C or 100 mU/ml of endo-β-galactosidase for 14 hours. KS digestion was confirmed by sequential digestion with 100 mU/ml of keratanase II for 3 hours and 100 mU/ml of endo-β-galactosidase for 14 hours and conversely with 100 mU/ml of endo-β-galactosidase for 14 hours and 100 mU/ml of keratanase II for 3 hours. This approach will generate four digestion products from the internal KS chain (unsulphated disaccharide, GlcNAcβ1,3Gal; fucosylated trisaccharide, Galβ1,2 [fucα1,3]GlcNAc6S; monosulphated disaccharide, Galβ1,4GlcNAc6S; and disulphated disaccharide, Gal6Sβ1,4glcNAc6S) and two digestion products that cap the KS chain (sialylated monosulphated trisaccharide NeuAα2,3Galβ1,4GlcNAc6S and disulphated trisaccharide NeuAα2,3Gal6Sβ1,4GlcNAc6S).

### Fluorotagging and FACE separation

Lyophilised enzyme-digested samples and predefined saccharide standards (Seikagaku Corporation) were reconstituted with the fluorescent tag, 5 μl of 12.5 mM 2-aminoacridone, in glacial acetic acid/dimethyl sulfoxide (3:17, vol/vol) and incubated at room temperature for 15 minutes. Five microlitres of 1.25 M sodium cyanoborohydride in distilled deionised water was added. Samples were incubated at 37°C for 16 hours. After tagging, 10 μl of 25% glycerol (20% vol/vol final concentration) was used to quench excess sodium cyanoborohydride. Electrophoresis was carried out for 80 minutes at 4°C as previously described [31].

### FACE gel imaging and quantitation

Gels were placed on a transilluminator light box fitted with a 312-nm light source. Fluorescent images were displayed using a GelDoc-It High CCD [charge-coupled device] Camera (12-bit depth; Ultra-Violet Products Ltd [UVP], Cambridge, UK), and the mean pixel density for each product band was quantified using LabWorks Software (UVP). For each gel, FACE product bands were identified by their coelectrophoresis with a range of predefined saccharide fluorotagged standards. Two image exposures were captured. The first exposure was used for quantitation as it had all of the pixels within a linear 12-bit depth range to provide baseline data, and the second exposure oversaturated pixel intensity to allow identification of less abundant structures. Accurate quantitation was achieved between 10 and 400 pmol of product.

Quality control was rigorously and routinely performed using a mixture of a known concentration of fluorotagged saccharide standards and enzyme-digested appropriate substrate controls. To control for gel-to-gel variation, one cadaveric cartilage sample was repeated when running multiple gels.

### Histology and immunohistochemistry

Cryosections were stained with haematoxylin and eosin and with toluidine blue for general histological assessment of cartilage and evaluation of metachromasia [3,4]. Immunostaining was performed using monoclonal antibodies (kindly provided by Professor Bruce Caterson, Cardiff University, UK) for chondroitin 4-sulphate (2-B-6), chondroitin 6-sulphate (3-B-3), and KS (5-D-4) [3,4]. Sections were initially treated with 25 mU/ml cABC, which generates 'stub' unsaturated disaccharides on the proteoglycan core proteins and also aids in unmasking of epitopes before incubating with primary antibodies. Sections were then labelled with biotin-conjugated horse anti-mouse immunoglobulin (Ig) G (which crossreacts with IgM; Vector Laboratories, Burlingame, CA, USA) before incubating with methanol and 0.3% (vol/vol) hydrogen peroxide to remove endogenous peroxidase activity. Labelling was amplified via avidin-peroxidase (Vectastain Elite ABC kit; Vector Laboratories) and demonstrated with diaminobenzidine as substrate. Sections that served as controls were treated with either normal mouse control IgG or phosphate-buffered saline alone, in place of primary antibodies.

## Results

### FACE analysis of hyaluronidase and chondroitinase digestion products

**Figure 1:**
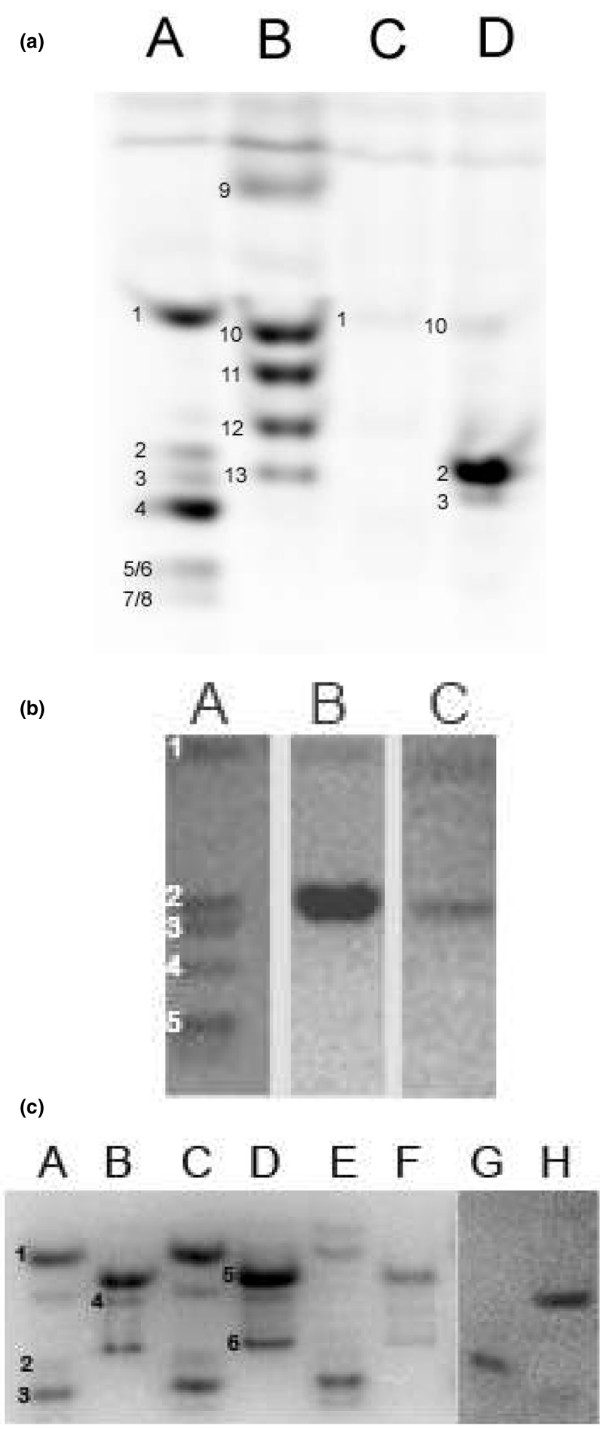
Fluorophore-assisted carbohydrate electrophoresis (FACE) analysis. **(a) **Representative FACE gel of hyaluronidase and chondroitinase digestion products from a 30-year-old cadaveric control. Lanes A and B contain a mixture of predefined fluorotagged saccharide standards. Lane A: (1) ΔDiHA, (2) ΔDi6S, (3) ΔDi4S, (4) ΔDi2S, (5) ΔDi4,6S, (6) ΔDi2,6S, (7) ΔDi2,4S, (8) ΔTri2,4,6S. Lane B: (9) *N*-acetylgalactosamine (GalNAc), (10) ΔDi0S, (11) GalNAc6S, (12) GalNAc4S, (13) 4-/6-sulphated GalNAc. Samples were digested with hyaluronidase (from *Streptococcus dysgalactiae*) (lane C) and chondroitinase ABC (lane D). **(b) **Representative FACE gel of chondroitin sulphate termini products from a 22-year-old cadaveric control (lane B) and a 22-year-old autologous chondrocyte implantation (ACI) patient (lane C). Lane A contains a mixture of predefined fluorotagged saccharide standards. Lane A: (1) GalNAc, (2) GalNAc6S, (3) GalNAc4S, (4) GalNAc4,6S, (5) GalNAc2,4,6S. **(c) **Representative FACE gel of keratanase II and endo-β-galactosidase digestion products obtained from full-depth cartilage of a 30-year-old cadaveric control (lanes E and F) and a 27-year-old ACI patient (lanes G and H). Lanes A to D contain a mixture of predefined fluorotagged saccharide standards. Lanes A to D: (1) Galβ1,2 [fucα1,3]GlcNAc6S, (2) Galβ1,4GlcNAc6S, (3) Gal6Sβ1,4glcNAc6S, (4) NeuAα2,3Gal6Sβ1,4GlcNAc6S, (5) GlcNAcβ1,3Gal, (6) GlcNAc6Sβ1,3Gal. ΔDi0S, unsulphated chondroitin sulphate disaccharide; ΔDi4S, chondroitin-4 sulphate disaccharide; ΔDi6S, chondroitin-6 sulphate disaccharide; ΔDiHA, hyaluronan disaccharide.

Figure 1a shows a representative FACE gel showing the coelectrophoresis of a known concentration of mono- and disaccharide fluorotagged standards alongside hyaluronidase and chondroitinase digestion products obtained from full-depth cartilage from the lateral femoral condyle from a 30-year-old cadaveric control. For total CS, there were ΔDi6S (88%), ΔDi4S (9%), and ΔDi0S (3%). No other disaccharide bands were observed. The quantities of total CS and HA disaccharides (ΔDiHA) in this biopsy were determined to be 1.778 μg/mg ± 0.005 standard deviation (SD) (*n *= 8) and 0.277 μg/mg ± 0.034 SD (*n *= 8), respectively. These data compare well with previously published data for adult articular cartilage [22,32]. We included this cadaveric cartilage sample when running multiple gels to control for gel-to-gel variation. To ensure that GAGs were not lost during processing, we performed enzyme digestions with appropriate substrate controls (data not shown).

**Figure 2:**
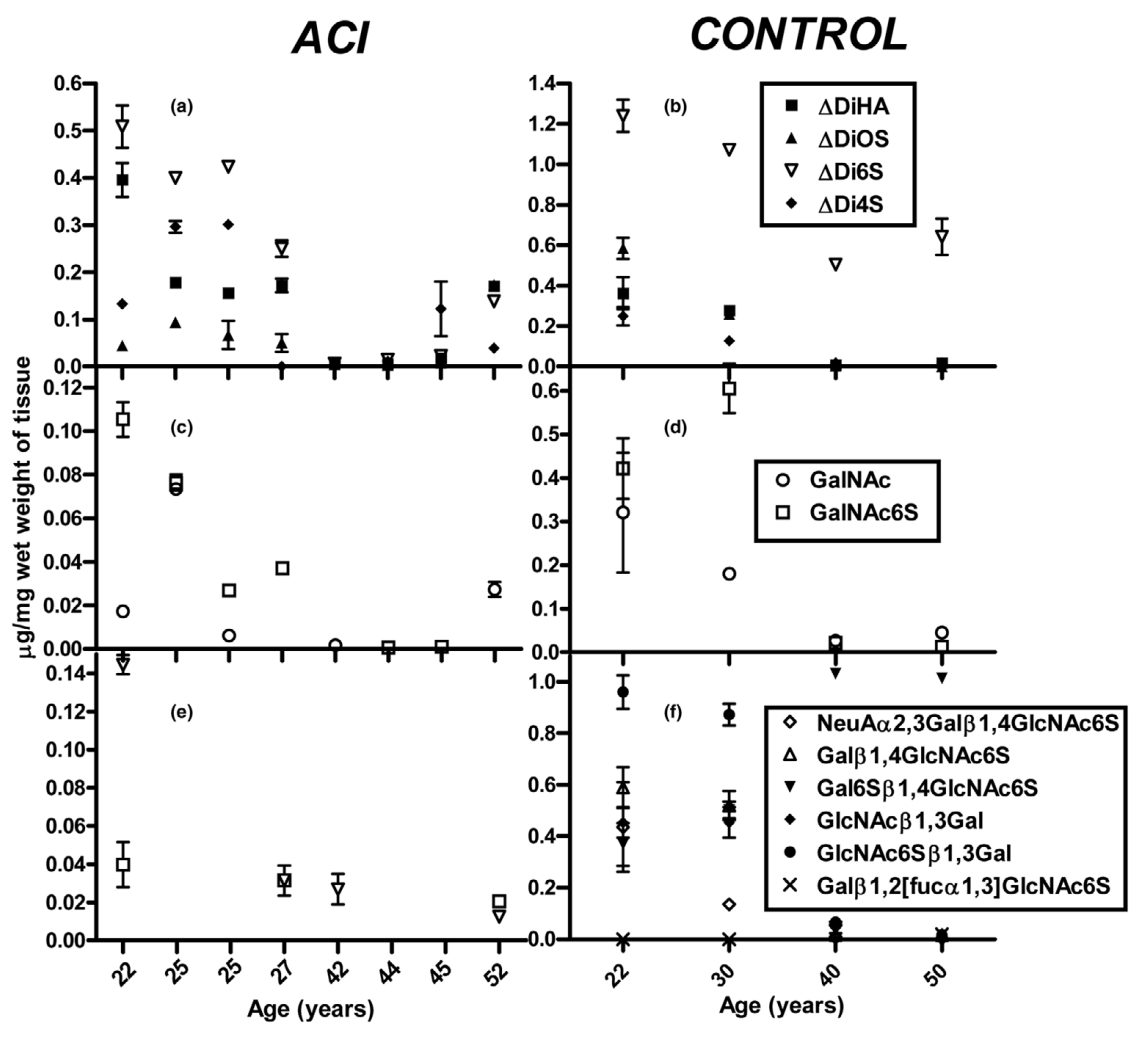
Fluorophore-assisted carbohydrate electrophoresis analysis of hyaluronidase, chondroitinase, keratanase II, and endo-β-galactosidase digestion products. Quantitation of levels of **(a,b) **hyaluronan and chondroitin sulphate (CS) disaccharides, **(c,d) **nonreducing terminal sugars of CS, and **(e,f) **keratan sulphate in autologous chondrocyte implantation (ACI) repair and cadaveric control tissues. Data are expressed as micrograms of disaccharide per milligram of wet weight of cartilage tissue. For each biopsy, the data are presented as the mean ± standard deviation of the replicate measurements. Differences between the ACI repair and cadaveric tissues were analysed using the Mann-Whitney *U *test (*p *< 0.05%). ΔDi0S, unsulphated chondroitin sulphate disaccharide; ΔDi4S, chondroitin-4 sulphate disaccharide; ΔDi6S, chondroitin-6 sulphate disaccharide; ΔDiHA, hyaluronan disaccharide; GalNAc, *N*-acetylgalactosamine.

The levels of HA disaccharides in both ACI and control groups followed a similar trend with respect to their decrease with increasing age (Figure 2a,b). The ACI group had an increased concentration of HA (*p *< 0.05%). In the ACI group, the ΔDi4S levels were similar to control whereas the ΔDi0S levels and ΔDi6S levels were lower (Figure 2a,b; *p *< 0.05%).

Figure 1b shows a representative FACE gel showing the separation of known concentrations of mono- and disaccharide fluorotagged standards alongside products of the analysis of the CS termini residues. The samples were obtained from full-depth cartilage from the medial femoral condyle from a 22-year-old ACI patient and an age-matched cadaveric control. For both the repair and control tissue groups, analysis of the terminal sugars of CS identified two different nonreducing termini: GalNAc and GalNAc6S (Figure 2c,d). A significant decrease in the concentration of both terminal sugars was detected with advancing age. In the repair tissue, there was at least twofold less total CS than in the control tissue.

### FACE analysis of keratanase II and endo-β-galactosidase digestion products

Figure 1c shows a representative FACE gel showing the coelectrophoresis of a known concentration of saccharide fluorotagged standards alongside keratanase II and endo-β-galactosidase digestion products obtained from full-depth cartilage from the lateral femoral condyle from a 27-year-old ACI patient and a 30-year-old cadaveric control. In the ACI repair tissue group, there was a significant reduction in all digestion products when compared with the control group (Figure 2e,f; *p *< 0.05%). Only two different digestion products from the internal KS chain were detected: Galβ1,4GlcNAc6S and Gal6Sβ1,4glcNAc6S. Both decreased with increasing age. We were unable to detect any terminal sugars. By contrast, in the control group, there was a significant increase in sulphation with increasing age and four different digestion products were detected (Figure 2f). The 40- and 50-year-old controls comprised mostly disulphated KS, whereas the 22- and 30-year-old controls comprised mostly un- or monosulphated KS. We detected the presence of one sialic-capped terminal residue, NeuAα2,3Galβ1,4GlcNAc6S, in the control group.

### Determination of the average chondroitin sulphate chain size by FACE analysis

**Figure 3:**
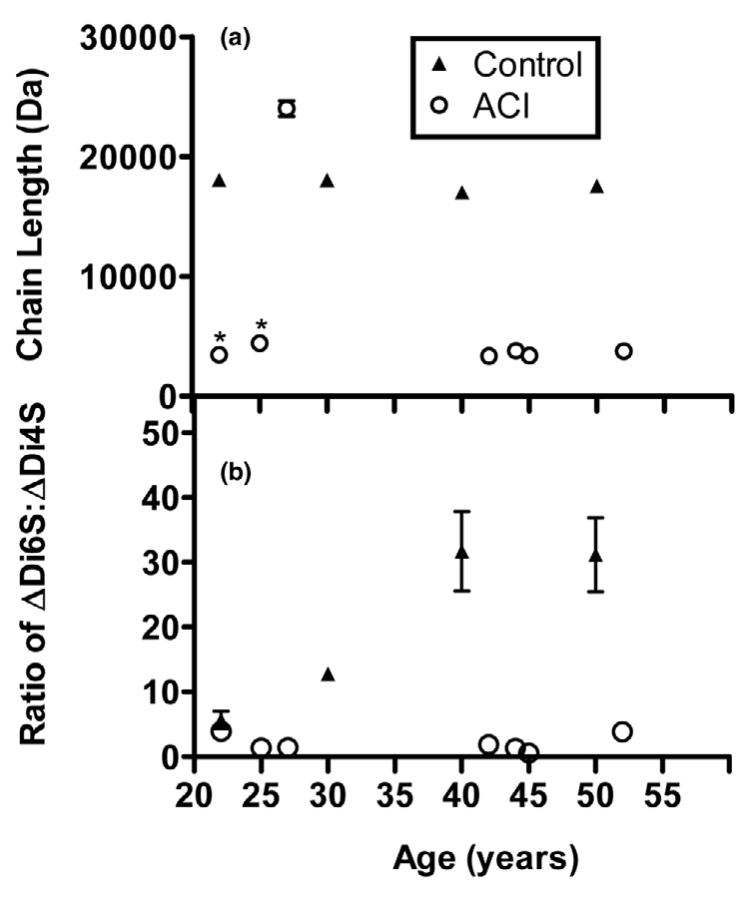
Determination of the averaged chondroitin sulphate chain size by fluorophore-assisted carbohydrate electrophoresis analysis and ratio of ΔDi6S/ΔDi4S. Comparison of **(a) **number averaged chondroitin sulphate chain size and **(b) **ratio of ΔDi6S/ΔDi4S in autologous chondrocyte implantation (ACI) repair and cadaveric control tissues. For each biopsy, the data are presented as the mean ± standard deviation of the replicate measurements. Differences between the repair and control tissue were analysed using the Mann-Whitney test (*p < 0.05%). ΔDi4S, chondroitin-4 sulphate disaccharide; ΔDi6S, chondroitin-6 sulphate disaccharide.

The value for the average CS chain length was determined by dividing the total fluorescence of the internal disaccharides by the total fluorescence of the nonreducing termini as previously described [33]. In the control group, the chain length was 18,208 Da ± 164 SD (Figure 3a). By contrast, CS chains in the repair tissue were significantly shorter but approximately the same with advancing age (4,003 ± 740 SD). Interestingly, the exception was the 27-year-old sample, which had the longest CS chains (24,905 Da).

### Ratio of ΔDi6S/ΔDi4S

In the control group, the ΔDi6S/ΔDi4S ratio increased with advancing age (Figure 3b), which is in agreement with previous studies [14,32]. In the repair tissue, the ΔDi6S/ΔDi4S ratio was significantly lower than in the control tissue and did not alter with advancing age.

### Proportion of chondroitin sulphate, hyaluronan, and keratan sulphate in the biopsies

**Figure 4:**
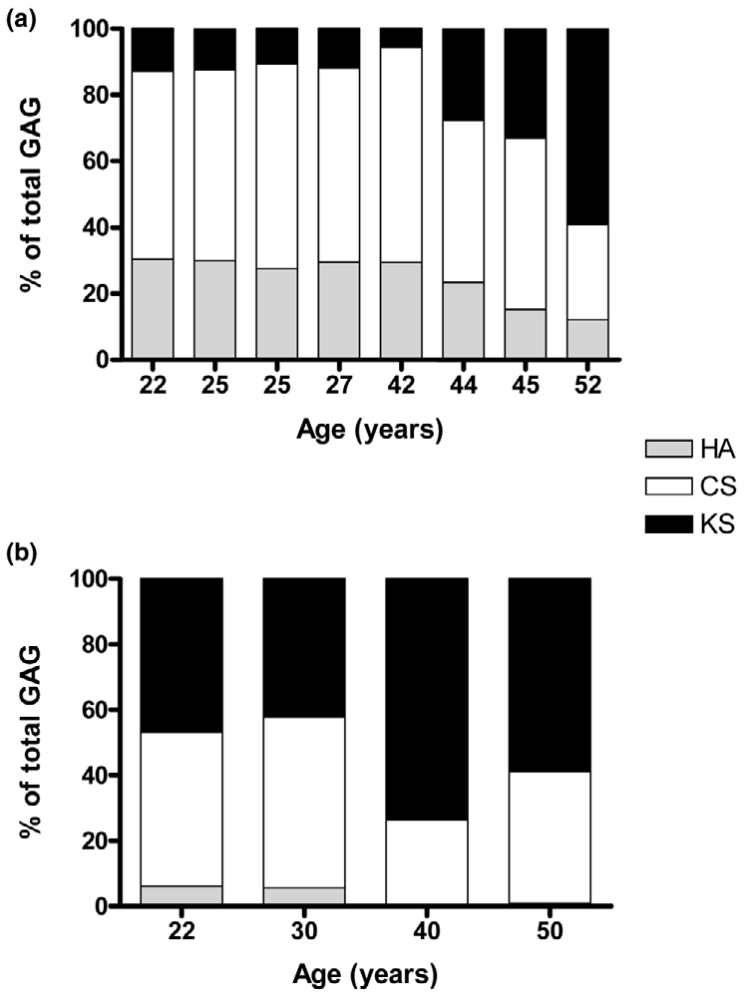
Proportions of chondroitin sulphate (CS), hyaluronan (HA), and keratan sulphate (KS) in the biopsies. Proportions of HA, CS, and KS in biopsies from **(a) **patients 1 year after autologous chondrocyte implantation and **(b) **controls. Each glycosaminoglycan (GAG), with its constituent disaccharide and monosaccharide component, is expressed as a total percentage of GAGs analysed by fluorophore-assisted carbohydrate electrophoresis.

FACE analysis revealed the differences in the proportions of HA, CS, and KS between the ACI repair and cadaveric control groups (Figure 4). Each GAG, with its constituent saccharide component, was expressed as a total percentage of GAGs analysed. In the repair group (Figure 4a), HA accounted for 20% to 30% of the total GAG, whereas in the control group (Figure 4b), HA accounted for approximately 6% of the total GAG. There was a greater proportion of CS than KS in the repair group. The inverse was true for the control group.

### Histology and immunohistochemistry

**Figure 5:**
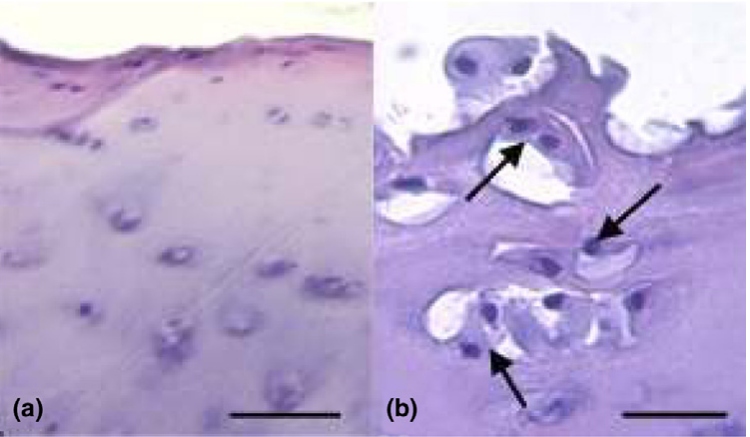
Control tissue stained with haematoxylin and eosin. Representative sections from the medial femoral condyle of **(a) **a 30-year-old and **(b) **a 50-year-old to illustrate the surface layer in cartilage taken. The arrows indicate chondrocyte clusters. The bars represent 25 μm.

**Figure 6:**
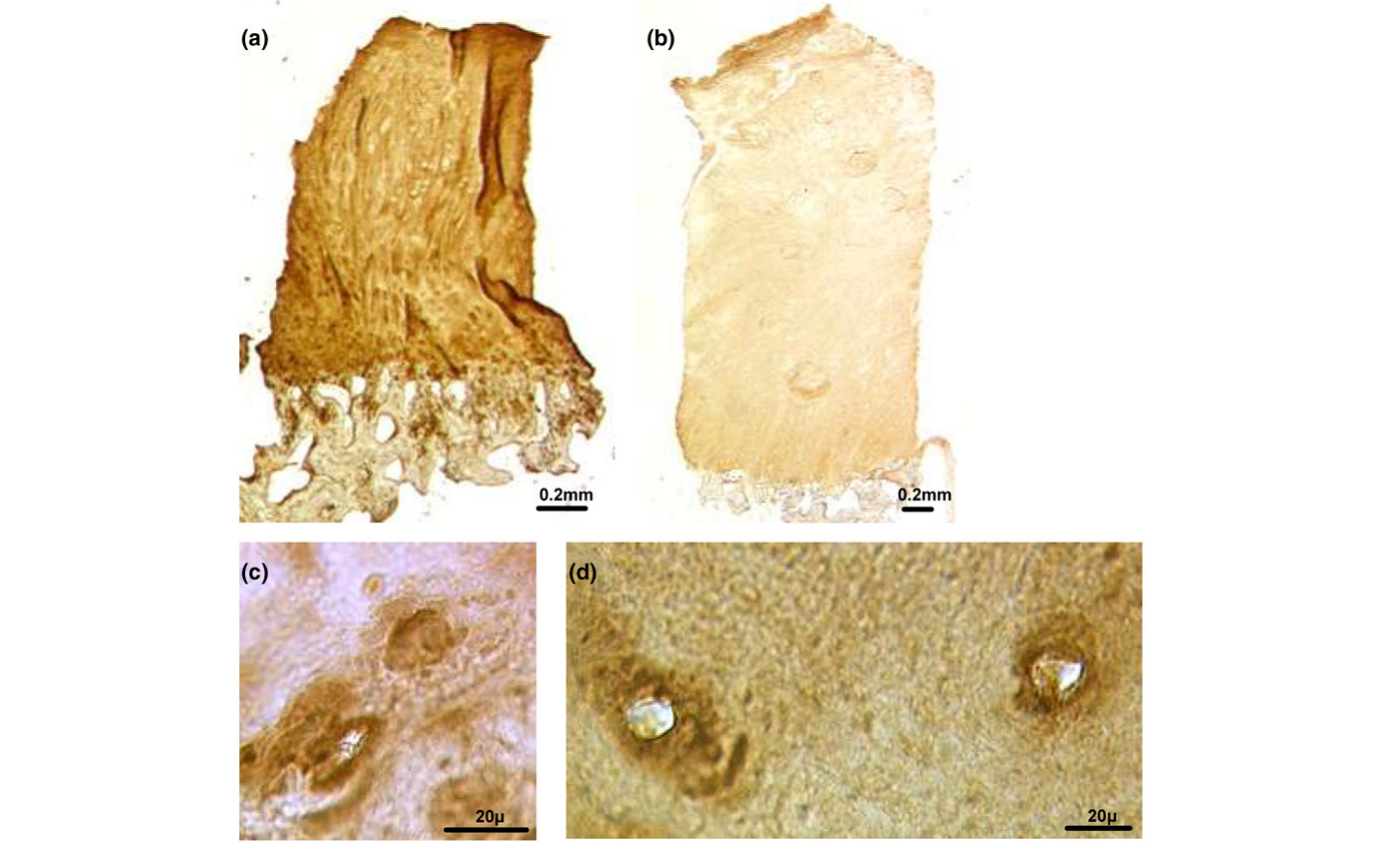
Immunohistochemistry to assess glycosaminoglycan distribution. Cartilage sections immunohistochemically stained for **(a,b) **chondroitin-4-sulphate and **(c,d) **chondroitin-6-sulphate. Repair tissue from autologous chondrocyte implantation (ACI)-treated patients with the highest **(a) **(22-year-old) and lowest **(b) **(52-year-old) levels of chondroitin-4-sulphate measured by fluorophore-assisted carbohydrate electrophoresis (FACE). Biopsies of cartilage from a 22 yr old ACI patient (c) and its age-matched control (d) immunostained for chondroitin 6-sulphate. The pattern of immunostaining in repair tissue from the ACI-treated patient **(c) **with the highest levels of chondroitin-6-sulphate as measured by FACE demonstrated a pericellular pattern, similar to that seen in the age-matched cadaveric control tissue but there was also more widespread matrix staining **(d)**.

Histology and immunohistochemistry were used to provide a baseline reference for comparison with FACE analysis. For the control group, the cartilage morphology was hyaline with a thickness of 2.15 to 2.8 mm. The sample from the 50-year-old contained some chondrocyte clusters and apparent degeneration of the articulating surface in comparison with that of the 30-year-old (Figure 5). For the repair group, the cartilage morphology was generally fibrocartilage (data not shown). Immunohistochemistry was performed to assess GAG distribution. The control cartilage was immunopositive for 4-sulphated CS (2-B-6), 6-sulphated CS (3-B-3), and KS (5-D-4) with a homogenous distribution through the matrix apart from in the deep zone, where it was more pericellular. There was no obvious difference with age. In the ACI biopsies, immunostaining was generally homogenous throughout for all GAGs, except in a few biopsies where it was pericellular in the deep zone. Figures 6a and 6b show representative sections of repair tissue from ACI patients with the highest and lowest levels of 4-sulphated CS as measured by FACE. There is a clear difference in intensity of immunostaining, reflecting this. FACE analysis demonstrated twofold less 6-sulphated CS disaccharide levels in biopsies from the youngest ACI patient (22-year-old) compared with the age-matched cadaveric control (22-year-old). Figures 6c and 6d show that there is a slight difference in staining intensity between these samples which is limited to a pericellular distribution in the ACI patient but which extends throughout the matrix generally in the control sample. These data illustrate that the intensity of immunostaining was generally comparable with the data obtained with FACE.

## Discussion

Currently, there is a lack of quantitative and qualitative biochemical data regarding the nature of ACI repair tissue, particularly in humans. This is partly due to the size of the tissue biopsy retrieved and the reluctance to disturb the repair site following surgical repair. We are in a relatively unique position of having access to repair tissue. In this study, we have used FACE to analyse GAG profiles in order to further our understanding of the composition of the repair tissue compared with age- and site-matched control cartilage.

We determined that FACE can assess as little as 5 μg of cartilage and detect GAG saccharides and nonreducing termini in the picomolar range. FACE has the additional benefit over standard GAG techniques in that it enables processing and imaging of a number of samples simultaneously. The results for FACE analysis and intensity of immunostaining were generally comparable. However, immunostaining cannot provide true quantitation as staining intensity will be affected by factors such as section thickness, antigen affinity, masking of epitopes, effectiveness of enzyme pretreatment, or stoichiometry. FACE thus offers huge advantages over immunostaining. For example, FACE enables an estimation of the number averaged GAG chain length. Our data demonstrate that, as an outcome measure, FACE can not only complement but also enhance existing histology and immunohistochemistry techniques for repair tissue analysis.

The most striking finding was that 1 year after ACI surgery the repair tissue contained half as much KS and CS as the control tissue. These observations support previously published studies [2-8] that have reported the fibrocartilage nature of ACI repair tissue 12 months after treatment. There could be a number of reasons to explain this finding. Firstly, cartilage remodelling is notoriously slow, with the half-life of aggrecan exceeding 3 years [9,18]. Secondly, the cells present in the defect may have a lower propensity for either specific GAG or proteoglycan synthesis. For ACI, the cells are expanded in culture for several passages *in vitro *prior to their implantation. It is unclear how this *in vitro *expansion affects the cell when it is placed into the cartilage defect. Thirdly, the GAGs that are synthesised at an early stage may not be retained within the repair tissue. They, along with their associated proteoglycan, may diffuse out of the small volume of surrounding extracellular matrix. Furthermore, there is likely to be insufficient collagen present to entrap the proteoglycans. This commonly occurs in the early phases of *in vitro *chondrocyte expansion as well as immediately after seeding chondrocytes onto three-dimensional scaffolds for tissue engineering articular cartilage [35,36].

In the repair group, the proportion of HA was higher than in the control group. Our values for the control group were similar to previous FACE studies of normal cartilage [22,33]. High concentrations of HA are generally found during development and the early stages of wound healing and repair [26,27]. Our observations suggest that the cells may be producing an HA-rich matrix to aid their migration and proliferation into the repair site. In the control group, the HA levels decreased with increasing age, which contrasted with a previous study using a radiosorbent assay that assessed HA levels in maturing and ageing tissue [20]. That study showed that HA levels increased from 20 to 30 years, reached a plateau from ages 30 to 70 years, and increased slightly after 70 years. It is difficult to compare our data with that study since we have such a small sample size.

In the repair group, the CS chains were considerably shorter. Variations in CS chain length can be induced during atypical proteoglycan synthesis. In addition, shorter chains have been linked with cartilage degeneration [14] but there is still a paucity of data from human subjects. These changes may be important for cartilage homeostasis. In the present study, CS content and the ratio of ΔDi6S/ΔDi4S were lower in the repair tissue. Functional implications of CS sulphation in cartilage have yet to be elucidated. However, changes in sulphation levels and patterns are known to be influenced by both mechanical loading and metabolic activity. During normal cartilage maturation, CS undergoes major changes in sulphation up to the age of 20 years; ΔDi6S content increases whilst ΔDi4S content decreases [14]. After the age of 20, both ΔDi6S and ΔDi4S content remain fairly constant but there are distribution changes within different cartilage zones. Unfortunately, we had insufficient ACI repair tissue available to perform a zonal analysis. It is important to emphasise that we have analysed only 1-year post-ACI biopsies from discrete regions and that the repair tissue profile may be very different at earlier or later time points. In this study, we detected two CS termini saccharides: galNAc and galNAc6s. Several other groups [17,31,32,37,38] have assessed the nonreducing termini residues in human CS by means of either biosynthetic or chemical techniques. None of the groups examined the CS termini from an equivalent age range (22 to 52 years) and consequently it is difficult to make direct comparisons with our data.

The KS digestion products identified in this study corresponded to those detected by previous investigations employing FACE [13]. The results obtained illustrate that there is less KS in ACI repair tissue and that it is more sulphated when compared with the control. Lower KS levels could be associated with the monolayer *in vitro *expansion of chondrocytes prior to ACI. It has been reported that KS synthesis decreases or stops when chondrocytes assume a fibroblastic morphology or when tissue is wounded [27,39]. However, it is more likely to be because the repair tissue is predominantly fibrocartilaginous, less like mature articular cartilage, and hence producing less KS. Furthermore, in articular cartilage, there is an increase in the proportion of KS with increasing age [9,39]. Hence, this tissue resembles younger and less mature or aged tissue. More sulphated KS chains could be the result of oversynthesis during tissue repair or they could indicate a level of fine modulation which may be necessary for tissue function. The sulphation levels of KS chains isolated from young cartilage (5 to 11 years old) have been shown to increase from 30%–50% to 80%–95% with advancing age [13]. We detected the presence of one sialic-capped terminal residue (NeuAα2,3Galβ1,4GlcNAc6S), which is in agreement with previously published work [13] that reported that sulphation of the terminal capping structures increases with maturity.

In this study, we have demonstrated that repair tissue formed after ACI has a lower concentration of GAGs, with very little KS, less chondroitin 6-sulphate, and shorter CS chains than site- and age-matched cadaveric articular cartilage. In contrast, ACI repair tissue has higher levels of HA than the controls. Thus, the repair tissue has some significant differences from normal adult articular cartilage.

## Conclusion

Currently, there is a paucity of biochemical data to determine whether ACI produces durable repair tissue. We are in a relatively unique position of having access to repair tissue 1 year post-ACI as well as age-matched cadavers. In the ACI repair tissue, there was an overall reduction in GAG content and the GAG populations differed from controls. These changes suggest that the composition of the repair tissue is not identical to mature articular cartilage. Further studies of long-term ACI patients are necessary to determine whether GAGs influence the long-term durability of repair tissue.

## Abbreviations

ΔDi0S = unsulphated chondroitin sulphate disaccharide; ΔDi4S = chondroitin-4 sulphate disaccharide; ΔDi6S = chondroitin-6 sulphate disaccharide; ΔDiHA = hyaluronan disaccharide; ACI = autologous chondrocyte implantation; cABC = chondroitinase ABC; CS = chondroitin sulphate; FACE = fluorophore-assisted carbohydrate electrophoresis; GAG = glycosaminoglycan; Gal = galactose; GalNAc = *N*-acetylgalactosamine; GlcA = glucuronic acid; GlcNAc = *N*-acetylglucosamine; HA = hyaluronan; Ig = immunoglobulin; KS = keratan sulphate; SD = standard deviation.

## Competing interests

The authors declare that they have no competing interests.

## Authors' contributions

AS carried out the experimental work, performed the statistical analysis, and drafted the manuscript. LDW helped with some of the experimental work. JBR took the ACI biopsies and helped to draft the manuscript. SR interpreted the immunohistochemistry data and helped to draft the manuscript. NJK designed and conceived the study, coordinated the project, and drafted the manuscript. All authors read and approved the final manuscript.
